# Barriers to equality, diversity and inclusion in research and academia stubbornly persist. So, what are we doing about it?

**DOI:** 10.1242/dmm.050048

**Published:** 2023-08-01

**Authors:** Lynne R. Prince, Sheila E. Francis

**Affiliations:** Division of Clinical Medicine, School of Medicine and Population Health, University of Sheffield, Beech Hill Road, Sheffield S10 2RX, UK

## Abstract

Despite an appetite for change, equality, diversity and inclusivity (EDI)-related issues continue to ripple through the world of research and academia, from inequity at the point of entry into education, through to lack of diversity and equality in senior roles. Many academic institutes and governments are taking action to solve these issues, and we welcome the growing number of inclusive practices in the science communication arena. Building from this, we – at the University of Sheffield, UK – have assessed our own situation, responded to pressures applied by research councils, and listened to our staff and student voice. Our new ‘One University’ initiative puts EDI on a par with research, innovation and education as a core university priority, and our Gender, Disability and Race Action Plans allow us to make measurable and impactful changes. Tackling EDI issues needs a collaborative approach, action at an institutional- or sector-wide level and clear commitment from senior leaders.

## Teaser

**Summary:** People from underrepresented groups are less likely to obtain research funding and secure senior academic positions. Systemic change requires organisational-level action, a diverse workplace, strong leadership and robust support networks in place.

Equality, diversity and inclusion (EDI)-related issues are ever prevalent in research and academia throughout all education and career levels, resulting in many underrepresented groups.

## What is the problem?

Equality, diversity and inclusion (EDI)-related issues are ever prevalent in research and academia throughout all education and career levels, resulting in many underrepresented groups (Box 1, Glossary). As of 2020/21, women comprise 49% of full-time academic staff in the UK and, yet, only 28% of professors are women. In the same period, ethnic minority (Box 1) staff of both genders accounted for 17% of all academic staff in the UK – closely mirroring population data showing that 18% of the UK self-identify within ethnic minority groups – but this reduces to 11% at professor level (HESA, 2022). Further evidence showed that researchers in STEMM (science, technology, engineering, mathematics and medicine) subjects with protected characteristics (Box 1; in this study, data were collected for age, disability, ethnicity and sex) continue to face barriers in obtaining funding and publishing their work (NIHR, 2022; UKRI, 2020). As an example, UK Research and Innovation (UKRI) funding award rates for principal investigators (PIs) are lower for women versus men, as are rates for ethnic minority applicants versus white counterparts (data from 2018–2019) (UKRI, 2020). Furthermore, awardees who are women and/or from ethnic minorities typically secure smaller grants (UKRI, 2020). A similar picture is seen for National Institute for Health and Care Research (NIHR) funding in the UK, where 12% of applicants from an ethnic minority background are successful in their funding applications, compared with 21% of white applicants (NIHR, 2022). Likewise, the proportion of NIHR funding award holders who are women reduces with increasing career stage, from 83% at the pre-doctoral stage, to 63% at the post-doctoral stage and 34% at the senior investigator level (NIHR, 2022). A study of research culture among new PIs in the UK, carried out by University College London and the University of Sheffield, found gender imbalance from the very beginning of an independent career (Acton et al., 2019). PIs who are women have lower starting salaries, smaller start-up packages and greater teaching load compared with men in equivalent positions (Acton et al., 2019). Greater transparency, positive actions and fairness around these career-influencing matters is keenly required.

**Box 1. Glossary**

**Underrepresented groups:** we use the term underrepresented groups to describe subsets of the population that hold a smaller percentage of senior roles in academia, particularly in STEMM subjects, compared with their representation in the general population.

**Ethnic minority:** in the UK, the term ethnic minorities refers to all ethnic groups except the white British group. White minority groups also exist, including traveller communities. Ethnic groups are categorised by ethnicity, i.e. a social identity based on common historical origins, and not race, i.e. a social construct describing people with shared physical characteristics.

**Protected characteristics:** these are characteristics that are protected by the Equality Act 2010, including age, disability, gender reassignment, marriage and civil partnership status, pregnancy and maternity, race, religion or belief, sex, and sexual orientation.

**Ethnicity awarding gap:** this refers to students from ethnic minority groups who enter the University with the same entry grades as white students but are on average awarded lower degree classifications.

**Gender pay gap:** this measures the difference in average earnings between women and men across the workplace.

**Socio-economic status:** a way of describing people based on their education, income and occupation.

**Intersectionality:** a concept recognising that protected characteristics and socio-economic factors do not exist independently in individuals. The overlapping, intersectional nature of this can amplify discrimination and inequity.

## National and international initiatives to fix the problem

UKRI and Advance HE (higher education) in the UK have recently reviewed challenges in the research and innovation sector, as well as the approaches taken to tackle these, on a national and international scale. The key findings are that interventions are mainly focused on gender equality or general EDI issues, but less so on characteristics, such as disability, religious inclusion, socio-economic status (Box 1) or age (Moody and Aldercotte, 2020; Guyan and Oloyede, 2020). The report indicates that successful approaches were often collaborative – across and within organisations – and had clear commitment from senior management. The Athena SWAN Charter framework to support gender equality in HE is adopted by a number of HE and research institutions across the world. But how can we use schemes like this to enact change?

A UK initiative launched in 2016 (announced in 2011), led by the Director General of Research and Development and the Chief Scientific Adviser at the Department of Health, prevented institutions that had not achieved an Athena SWAN silver award from being shortlisted for NIHR Research Centre funding (Ovseiko et al., 2020). The impact of this was stark: it resulted in a tenfold increase in Athena SWAN awards and, therefore, greater support for women in research. This was associated with a rise in the number of women in mid-level leadership positions at research centres (24% in 2016, up from 8% in 2011) and increased the percentage of women in senior director positions (15% in 2016, up from 10% in 2011) (Ovseiko et al., 2020). Furthermore, there was an increase in the proportion of NIHR funding obtained by women (Ovseiko et al., 2020). The national ‘R&D People and Culture Strategy’ developed in 2021, sets out the UK Government's ambition to build the research and innovation workforce in a positive and inclusive culture (Department for Business, Energy & Industrial Strategy, 2021). This document prompted organisations such as UKRI to further develop EDI strategies for the research and innovation sector that focus on respect, valuing different people and ways of thinking, and allows people to be their ‘real-self’ (UKRI, 2022).

Looking beyond the UK, the National Institutes of Health (NIH) in the USA have recently published their 5-year strategic plan for Diversity, Equity, Inclusion and Accessibility (DEIA), which is set to enact structural and cultural change within the workforce and, perhaps more uniquely, to advance DEIA through the types of research they fund (NIH, 2023). In 2019, the League of European Research Universities (LERU), with over 23 member institutions across Europe, published their EDI position paper (LERU, 2019). The paper emphasises the importance of acknowledging bias in the workplace – especially for senior leaders, who typically do not belong to underrepresented groups and may, therefore, lack an appreciation of the barriers faced by others. It also highlights the need for communication, commitment and action from the highest levels of leadership, in order to change institutional culture (LERU, 2019).

[…] wellbeing and EDI has to be everyone's responsibility, and we need a collective appetite for change.

## What are we doing in Sheffield?

As of 2023, the University of York, University College London and Queen's University Belfast in the UK each have three department-level Athena SWAN Gold Awards – the highest of the award categories – in recognition of their excellent gender equality initiatives and practice. Like these institutions, we have pushed forward on EDI issues for several years, embracing initiatives like the Athena SWAN Charter. We have assessed our own situation, listened to our staff and student voice, and responded to the pressures applied by research councils, such as UKRI. Arguably, the most impactful change taking place at the University of Sheffield is to place workplace culture, wellbeing and EDI – via the sector leading ‘One University’ pillar (https://www.sheffield.ac.uk/vision/our-pillars/one-university) – on an equal standing with research, innovation and education, which sends a message about how important this is. Bringing wellbeing and EDI together is key, since tackling inequality with support systems focused on wellbeing can be quicker and more effective (Nicholls et al., 2022). This initiative needs serious commitment and action at an institutional/sector-wide level and to this end, we have One University Directors at department, faculty and university level, who are turning general wellbeing and EDI goals into actions with measurable outcomes and enacting systemic change. Furthermore, One University Committees are made up of a diverse group of academics and professional staff who share their lived experiences of barriers in academia, define actions that are required and judge the success of initiatives they help to implement. Although, for this to work, wellbeing and EDI has to be everyone's responsibility, and we need a collective appetite for change.

In our commitment to gender equality, we formed a programme to address structural barriers to equal opportunities and progression. These actions include improvements in recruitment processes, transparent allocation of senior leadership roles and career mentoring. We also conducted a review of how pastoral type roles tend to disproportionally fall to women and how this impacts research performance in academic staff at the University of Sheffield. Moreover, we have implemented a number of support networks, as well as targeted financial support, for parents, carers and people undergoing fertility treatment or experiencing menopausal symptoms.

As the UKRI investigation revealed, most efforts to address EDI issues have focused on gender. Therefore, we have also developed a broader series of Action Plans consisting of aims and actions that are continually reviewed and updated via a publicly available dashboard. These include Action Plans for Disability Equality (https://cc.sheffield.ac.uk/hr/disability/disability-equality-strategy-and-action-plan/), Race Equality (https://cc.sheffield.ac.uk/hr/race/race-equality-strategy-and-action-plan/), LGBT+ Equality (https://cc.sheffield.ac.uk/hr/lgbt/lgbt-equality-strategy-and-action-plan/) and Belief, Non-belief and Religion (https://cc.sheffield.ac.uk/hr/religion/religion-belief-and-no-belief-action-plan/). Within the Disability Equality Action Plan, we aim to understand the spectrum of seen and unseen disabilities, in order to build an equitable and inclusive culture, develop flexible learning and teaching models, and to create an accessible campus. Planned actions include involving people with disabilities at all stages in the design of new buildings, developing mandatory training programmes to enhance both the understanding of disability and the support that is needed, providing a centrally funded scheme to allow sports clubs and other student activity groups to make adaptations to aid inclusion, and hiring staff disability advisors. We are also considering how adjustments for disability can be made to career progression processes, ensuring staff have the individualised support they need to progress. This could include devising flexible working hours and providing specialised equipment or software.

Our Race Equality Action Plan aims to improve inclusion, and the sense of belonging for staff and students, by widening access for students from ethnic minority groups, reducing the ethnicity awarding gap (Box 1), increasing diversity of staff and supporting the career progression of staff from ethnic minority groups. Planned actions include better support for student progress and staff career development, staff mentoring schemes, staff networks, robust anti-racism and anti-unconscious bias training, and clear reporting mechanisms for racist behaviour. The importance of involving individuals from underrepresented communities throughout the development of these action plans cannot be emphasised enough.

**Fig. 1.**
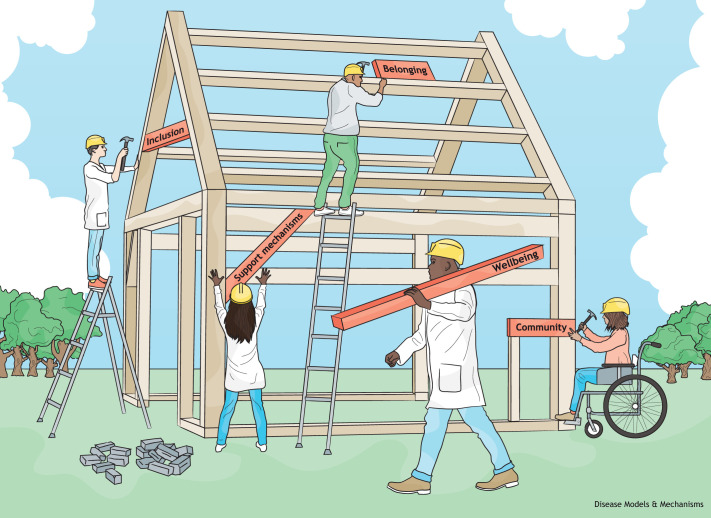
**Building an inclusive workplace environment.** Tackling systemic barriers in Equality, Diversity and Inclusion requires organisational level investment to build an environment that fosters a sense of inclusion, belonging and community, with wellbeing at the forefront of these efforts and tailored support mechanisms being an integral tool. This image is by neilsmithillustration.co.uk and published under the CC-BY 4.0 license for this article.

Since barriers are often intersectional (Box 1) and not mutually exclusive, we found – as the individual strategies evolved and delivery plans were introduced – duplication in areas, such as training and improving disclosure of equality data since, without these data, it is difficult to know what changes are needed or to monitor the effects of initiatives that are put in place. This required us to bring together actions that were common to multiple action plans to ensure they can be coordinated where possible. For example, improving career promotion processes featured in every action plan. Our university-wide Academic Career Pathway Framework is being modified to enable the delivery of combined actions specifically related to protected characteristics. This process highlighted that we must tackle the systemic barriers that are common to all EDI issues and, to this end, we have recently launched a Staff Code of Conduct, with EDI as a central theme. The Code sets standards of behaviour to help create a collaborative and inclusive workplace, to maintain responsible and ethical conduct, and to clearly outline reporting processes – for instance, via the online Report+Support portal. We strongly advocate to speak out against or to report harassment and bullying. This is because inclusion is firmly embedded within wellbeing, the environment and a sense of belonging, requiring us to nurture feelings of acceptance, connection and value (Fig. 1). At Sheffield, we also run formal and informal mentor schemes to support development and wellbeing for colleagues at all stages of their career, and help provide solutions for those that feel they are facing EDI barriers. To monitor this, as well as career development opportunities and fairness around EDI, we have launched a Staff Survey that allows us to make reactive institution-wide improvements to working practices based on what staff identify as issues and not what the institution *believes* are the problems.

How exactly will we know we have made a difference? Having clear measurable outcomes embedded into action plans keep us accountable in our intention to make a change. It will take time and will be challenging to see whether we have changed beliefs or practices. However, we can quantify changes to the gender pay gap (Box 1), the ethnicity awarding gap and staff recruitment and retention, as well as monitor the Report+Support portal metrics.

## Addressing EDI issues in scientific communication

An obvious solution to aid career progression for people from underrepresented groups in academia is avoiding bias in scientific publishing and communication (Dewidar et al., 2022). In 2020, the Royal Society of Chemistry launched the *Joint Commitment for action on equality and diversity in publishing*, which has now been joined by over 50 publishing organisations – including The Company of Biologists, publisher of Disease Models and Mechanisms (DMM) – with a combined portfolio of over 15,000 journals (Royal Society of Chemistry, 2020). The joint commitment sets minimum standards that include integrating inclusion and diversity into publishing activities, by understanding the diversity of authors, editors and reviewers, or lack thereof. Publishers, such as The Company of Biologists, have begun to acknowledge the barriers that authors, editors and reviewers from underrepresented communities experience, and have taken actions to address them (https://www.biologists.com/about-us/edi/). The global academic publisher Sage recognises the challenges faced by neurodiverse authors and offers 1:1 support throughout the publication process (Lowenstein, 2021). Perhaps, further work around alternative ways to publish research – for instance, via more visual and audio material – may also benefit neurodiverse researchers and readers.

Conferences should be a platform for all researchers to learn, disseminate findings and to network, irrespective of their caring responsibilities, disability or other needs. Conference organisers are beginning to understand the difficulties faced by some underrepresented groups in attending meetings and, therefore, in benefitting from the associated career development opportunities, although progress here is slow. Societies paving the way include the Zebrafish Disease Models Society, who have an EDI Committee and included a dedicated EDI session in their recent meeting programme. To make advances more broadly, all meeting organisers should have a diverse planning committee that represents researchers of different backgrounds and needs. It would be advantageous if organisers surveyed all participants for accessibility needs at the point of registration, and appointed an ‘Accessibility Chair’, who could be a point of contact for attendees with accessibility requirements (https://www.asbmb.org/asbmb-today/careers/111221/conferences-for-all). It is also possible, if not typical, to find childcare services at conference venues – although, even when available, the cost can be prohibitive. The Biophysical Society offers Family Care Grants of up to $500 to support individuals who require care for dependents in order to attend a conference, which is a step in the right direction.

## Outlook

Promoting diversity is relevant not just to researchers but also to participants in research, particularly in clinical trials. By not having a diverse range of participants in clinical research, results may not be applicable to the broader population, and clinicians may be unable to effectively treat certain patients if there is no evidence the intervention is efficacious in that particular group. The NIHR-INCLUDE project has produced guidance around what an under-served group is, and defined intervention points to improve inclusion in health and care research (NIHR, 2020). The US Food and Drug Administration (FDA) has issued guidance to industry to include more participants from underrepresented racial and ethnic groups in clinical trials (https://www.fda.gov/news-events/press-announcements/fda-takes-important-steps-increase-racial-and-ethnic-diversity-clinical-trials). To this end, they recommend industrial sponsors develop Race and Ethnicity Diversity Plans. Furthermore, as stewards of the largest public investment in biomedical and behavioural research in the USA, it is within the power of the NIH to advance DEIA through research; as a result, one of their objectives is to focus on supporting health research that is intentionally inclusive of intersectional characteristics, including race, ethnicity, sex, sexual orientation, gender identity, age, language, abilities, socio-economic status and geographic region (NIH, 2023). Bodicoat et al. make recommendations to help improve inclusion in clinical research, which include improving cultural sensitivity of clinical trial staff via training, diversifying advisory panels and increasing recruitment of staff from underrepresented groups (Bodicoat et al., 2021).

Recognising the value of diverse individuals and providing tailored support mechanisms will lead to unbiased career development and progression that benefits scientific research and academia as a whole.

Increasing diversity among our scientific workforce and educating leaders in decision-making positions about barriers faced and actions required will inevitably improve how we conduct all research relating to human health. The power of leading by example and being a positive role model cannot be underestimated. It is also worth stressing the importance of access to a diverse network of mentors who can provide tailored mentorship – since ‘one size does *not* fit all’ when it comes to support. We believe that, for mentoring to be truly effective, there also needs to be sponsorship, i.e. someone who can help define career strategies, create opportunities and be an advocate. If you are an early- or mid-career researcher and feel you are facing EDI barriers, look to a mentor or manager for support. If that support is not there, reach out to someone who could share how they have overcome their own challenges. Ultimately, tackling systemic barriers in EDI requires institutional-level investment in wellbeing, the environment and workplace culture. Recognising the value of diverse individuals and providing tailored support mechanisms will lead to unbiased career development and progression that benefits scientific research, and academia as a whole.
